# Draft Genome Sequence of *Pseudomonas fragi* Strain DBC, Which Has the Ability To Degrade High-Molecular-Weight Polyaromatic Hydrocarbons

**DOI:** 10.1128/genomeA.01347-17

**Published:** 2017-12-07

**Authors:** L. Paikhomba Singha, Rhitu Kotoky, Piyush Pandey

**Affiliations:** Department of Microbiology, Assam University, Silchar, India

## Abstract

*Pseudomonas fragi* strain DBC was isolated from crude oil-contaminated soil. The genome of *P. fragi* DBC is comprised of 5,072,304 bp with 54.09% GC content. Genes for degradation of polyaromatic hydrocarbons were found in the genome, in addition to genetic elements for related physiological functions such as chemotaxis, detoxification, and quorum sensing.

## GENOME ANNOUNCEMENT

Among various species of *Pseudomonas*, *P. fragi* is relatively less known for its role in biodegradation of hydrocarbons, although it has been reported for its plant growth-promoting characteristics (1, 2). *P. fragi* strain DBC was isolated from a crude oil-contaminated soil sample in northeast India. It was able to utilize high-molecular-weight polyaromatic hydrocarbons such as pyrene and benzopyrene as the sole sources of carbon and energy. To determine the complete genome sequence of *P. fragi* DBC, whole-genome shotgun sequencing was done using one Illumina paired-end library with an average insert size of ~400 bp. The paired-end sequencing libraries were prepared using the Illumina TruSeq Nano DNA library prep kit. The DNA was fragmented by Covaris M220, which generates double-sided DNA (dsDNA) fragments with 3′ or 5′ overhang. The fragments were then subjected to end repair followed by adapter ligation to the fragments. The products were PCR amplified with the index primer as described in the kit protocol and sequenced using Next Seq 500. The sequenced raw data were processed to obtain high-quality clean reads using Trimmomatic v0.35 to remove ambiguous reads, adapter, and low-quality sequences. These reads were trimmed using a quality score threshold of 20 and a length cutoff of 20 bp. Reference-guided assembly of the sample was performed using SAMtools. The procedure for genome annotation was done by Rapid Annotations using Subsystems Technology (RAST) server and NCBI prokaryotic genome annotation pipeline (https://www.ncbi.nlm.nih.gov) (3). The rRNA and tRNA genes were predicted and annotated using RNAmmer (4) and tRNAscan-SE (5), respectively. The genome of *P. fragi* DBC consists of one circular chromosome, 5,072,304 bp (54.09%, GC content).

The first version of annotation includes 3,993 protein-coding genes, 72 tRNA genes, and 25 rRNA genes. Genes involved in degradation and metabolism of aromatic hydrocarbons were found, e.g., *nod*, *nahR*, *nahA-F*, *catA*, *kshA*, *hsaC*, etc. This strain also carries the sulfur dioxygenase gene *sox* to remove covalently bound sulfur from dibenzothiophene (DBT). The strain DBC possesses several other genes required for adaptation to stress, signaling, and membrane transport system. Biofilm genes (*algc*), quorum-sensing genes, chemotaxis genes (*cheA*, *cheZ*, *cheY*, *fliN*, *mcpB,* etc.), and detoxifying genes *gstA* (glutathione-*S*-transferase) were also present.

### Accession number(s).

The complete genome sequence of *P. fragi* DBC has been deposited in GenBank under accession no. CP021986.
